# Supplementary material for: Propagation of a De Novo Gene under Natural Selection: Antifreeze Glycoprotein Genes and Their Evolutionary History in Codfishes
Source: Genes (Basel). 2021 Nov 9;12(11):1777. doi: 10.3390/genes12111777 (PMC8622921; doi:10.3390/genes12111777)
Supplement: Supplementary file 1 [file genes-12-01777-s001.zip › genes-1413316-supplementary.pdf]

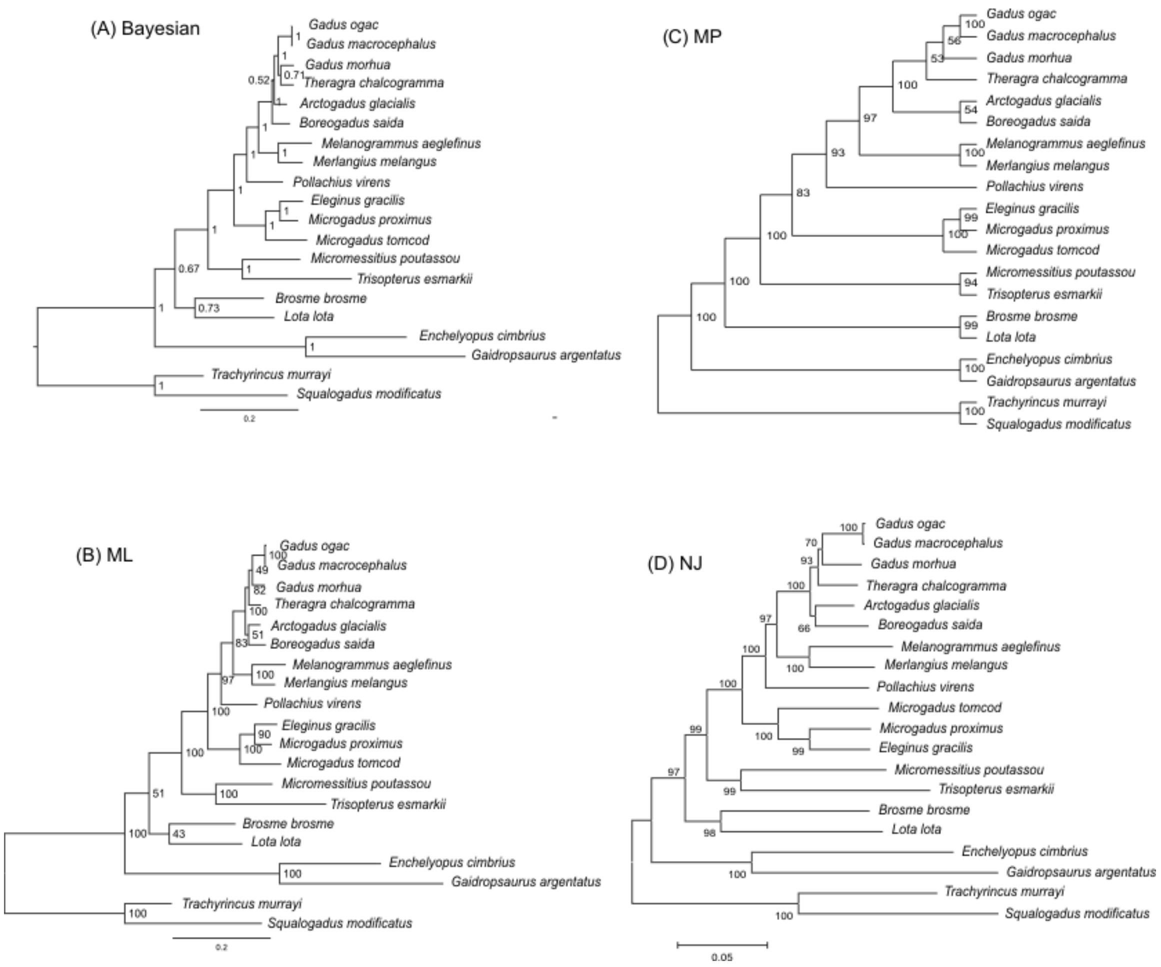

Figure S1 **Gadid phylogenetic trees** obtained by (A) Bayesian analysis, (B) Maximum Likelihood (ML) analysis, (C) Maximum Parsimony (MP) analysis, and (D) Neighbor – Joining (NJ) analysis of 18 gadid species using complete mitochondrial COI and ND2 gene sequences, and rooted with two gadiform species from family Macrouridae, *Squalogadus modificatus* and *Trachyrincus murrayi*. Numbers at branch nodes indicate the posterior probabilities on Bayesian tree, and the percentage support values (%) from 1000 bootstrap replications of ML, MP and NJ analyses on their respective tree.

Table S 1 Species and month and locality of sample collection*, and GenBank Accession numbers for COI sequences.

**Subfamily**<br>Species	Collected season	Sampling locality	GenBank Accession numbers (COI)
**Gadinae**			
Arctogadus glacialis	July	Uummannaq, W. Greenland	MK011286
Boreogadus saida	October	Spitzbergen	MK011278
Eleginus gracilis	March	Kotzbue, AK	MK011279
Gadus macrocephalus	March/August	Bogosloff Isld/Cook Inlet, AK	MK011275
*Gadus morhua***	August	Copenhagen Harbor	MK011280
*Gadus ogac***	March/August	Labrador/Disco Bay, W. Greenland	MK011287
Melanogrammus aeglefinus	April	Tromsoe fjord, Norway	MK011282
Merlangius merlangus	April	Tromsoe fyord, Norway	MK011283
Microgadus proximus	August	Cook Inlet, AK	MK011274
Microgadus tomcod	January	Shinnecock Bay, NY	MK011276
Micromesistius poutassou	April	Tromsoe fjord, Norway	MK011288
Pollachius virens	April	Tromsoe fyord, Norway	MK011284
Theragra chalcogramma	March	South Bering Sea	MK011277
Trisopterus esmarkii	April	Tromsoe fjord, Norway	MK011285
**Lotinae**			
Brosme brosme	September	Tromsoe fjord, Norway	MK011273
Lota lota	April	Oneida Lake, New York	MK011281
**Gaidropsarinae**			
Enchelyopus cimbrius	April	Finnmarch, Norway	MK011289
Gaidropsarus argentatus	October	Scoresbysund, East Greenland	MK011290

*Tissues and blood were collected from each species.

**Summer (August) *Gadus morhua* and *Gadus ogac* specimens exhibit low levels of antifreeze activity. For AFGP purification, early spring serum sample of *G. ogac* (Labrador coast) from our lab inventory was used. No winter serum samples were available for *G.morhua*.

Table S2 Characteristics of all AFGP genes and pseudogenes in three gadids

AFGP gene or pseudogene	CDS Length (bp)	Signal peptide	C-terminus non-tripeptide residues	Numbers of polyprotein cleavage sites		
				R	K	RAAR
Bs AFGP1ψ	~1.7k	Yes	RVCVCVCV*	4	2	0
Bs AFGP2	766	Yes	AAVL*	3	0	0
Bs AFGP3	603	Yes	AAVL*	1	1	0
Bs AFGP4	1791	Yes	AAVF*	2	4	0
Bs AFGP5	~2k	Yes	AAVF*	0	4	0
Bs AFGP6ψ	~800	No	AAVF*	1	3	0
Bs AFGP7ψ	959	Yes	AAVS*	1	3	0
Bs AFGP8	2256	Yes	AVF*	5	0	9
Bs AFGP9	2085	Yes	AVF*	2	0	9
Bs AFGP10	~2.7k	Yes	AVF*	1	0	5
Bs AFGP11	1254	Yes	AVF*	5	0	4
Bs AFGP12	1737	Yes	AVF*	5	0	7
Bs AFGP13	2052	Yes	AVF*	5	0	8
Bs AFGP14ψ	1288	No	AVF*	5	0	4
Bs AFGP15	1563	Yes	AVF*	6	0	5
Bs AFGP16	~1.2k	Yes	in gap	1	0	1
Gm AFGP1ψ	~2.6k	Yes	RVCVCVCV*	4	2	0
Gm AFGP2	529	Yes	AAVL*	2	1	0
Gm AFGP3	595	Yes	AAVL*	1	0	0
Gm AFGP4	637	Yes	AAVL*	1	1	0
Gm AFGP5	1566	Yes	AAVL*	4	0	0
Gm AFGP6	~2.8k	Yes	AAVF*	0	8	0
Gm AFGP7ψ	698	Yes	AAVS*	0	0	0
Mt AFGP1	796	Yes	AVF*	0	1	0
Mt AFGP2	~2.2k	Yes	AAVF*	0	1	0
Mt AFGP3	~4.5k	Yes	KL*	0	0	50
Mt AFGP4ψ	723	No	AAVS*	1	0	0

Notes: CDS, coding sequence. Pseudogenes are denoted by 'ψ' at the end of the gene name. The lengths of the complete genes are accurate to base pair (bp), and the estimated lengths of the incomplete genes (have gap in assembly) are indicated by ~. The symbol '*' in the column 'C-terminus non-tripeptide residues' indicates the stop codon. Amino acid is represented by single letter. The numbers in the last three columns represent the copy number of each type of polyprotein cleavage sites.

Table S3 . Comparison of amino acid (%) in mature AFGPs and AFGP gene (conceptual translation) in *B. saida*

	Size isoforms	Thr	Pro	Ala	Arg	Lys
Mature AFGPs*	AFGP1-5	29.8	1.2	67.0	1.2	0
	AFGP6	26.1	12.3	61.0	0.6	0
	AFGP7,8	26.6	14.1	58.6	0.7	0
AFGP genes		30.0	10.1	56.6	3.0	0.3

*Amino acid composition of mature AFGPs based on Chen et al 1997 {Chen, 1997b #41}.

Table S4 Codon usage frequencies of three residue positions in the tripeptide repeats of all *AFGPs* in three gadids

	Tripeptide 1st residue (%)							Tripeptide 2nd residue (%)							Tripeptide 3rd residue (%)			
	Thr	Thr	Thr	Thr	Arg	Lys	Ala	Ala	Ala	Ala	Ala	Pro	Pro	Pro	Ala	Ala	Ala	Ala
	ACA	ACT	ACC	ACG	AGA	AAA	GCT	GCA	GCT	GCC	GCG	CCA	CCT	CCG	GCA	GCT	GCC	GCG
Bs_AFGP2	39.6	25.0	31.3	0.0	4.2	0.0	0.0	16.7	0.0	56.3	12.5	8.3	0.0	6.3	14.6	2.1	81.3	2.1
Bs_AFGP3	32.3	41.9	19.4	0.0	3.2	3.2	0.0	39.3	0.0	7.1	21.4	17.9	0.0	14.3	17.2	0.0	82.8	0.0
Bs_AFGP4	51.2	27.7	15.1	0.6	1.2	2.4	1.8	32.5	0.6	31.3	27.1	4.8	0.0	3.6	20.5	4.2	75.3	0.0
Bs_AFGP5	31.3	46.9	0.0	0.0	0.0	9.4	12.5	45.5	0.0	3.0	0.0	48.5	3.0	0.0	28.1	0.0	71.9	0.0
Bs_AFGP6	22.9	54.3	2.9	0.0	2.9	8.6	8.6	47.2	0.0	0.0	0.0	52.8	0.0	0.0	25.7	2.9	71.4	0.0
Bs_AFGP8	40.7	40.7	4.2	0.0	13.6	0.0	0.9	39.0	0.0	10.3	22.1	11.7	0.0	16.9	48.6	9.5	41.9	0.0
Bs_AFGP9	45.6	39.0	4.6	0.0	9.7	0.0	1.0	41.2	0.0	13.4	22.2	10.8	0.0	12.4	41.5	10.4	48.2	0.0
Bs_AFGP10	45.2	38.5	4.8	0.0	10.6	0.0	1.0	39.0	0.0	15.2	21.0	11.4	0.0	13.3	41.7	11.5	46.9	0.0
Bs_AFGP11	36.4	44.4	5.1	0.0	13.1	0.0	1.0	30.6	0.0	14.3	22.4	18.4	0.0	14.3	57.3	5.2	37.5	0.0
Bs_AFGP12	41.7	42.3	3.2	0.6	12.2	0.0	0.0	35.5	0.0	9.2	23.7	15.8	0.0	15.8	48.7	11.3	40.0	0.0
Bs_AFGP13	42.4	40.0	4.0	0.0	12.8	0.8	0.0	38.7	0.0	8.9	21.8	13.7	0.0	16.9	40.8	12.0	47.2	0.0
Bs_AFGP14	39.2	40.8	7.5	0.0	10.0	0.8	1.7	38.7	0.8	13.4	16.8	13.4	1.7	15.1	44.2	7.5	47.5	0.8
Bs_AFGP15	37.5	41.2	7.4	0.0	11.0	1.5	1.5	40.6	0.0	13.3	17.2	10.2	1.6	17.2	41.4	10.5	48.1	0.0
Bs_AFGP16	48.6	37.8	5.4	0.0	5.4	2.7	0.0	33.3	0.0	8.3	33.3	16.7	0.0	8.3	45.9	13.5	40.5	0.0
**Bs Average**	**39.6**	**40.0**	**8.2**	**0.1**	**7.8**	**2.1**	**2.1**	**37.0**	**0.1**	**14.6**	**18.7**	**18.2**	**0.4**	**11.0**	**36.9**	**7.2**	**55.7**	**0.2**
Gm_AFGP2	42.9	28.6	9.5	0.0	9.5	4.8	4.8	30.0	5.0	25.0	15.0	20.0	0.0	5.0	19.0	0.0	81.0	0.0
Gm_AFGP3	40.0	26.7	30.0	0.0	3.3	0.0	0.0	36.7	3.3	30.0	16.7	6.7	3.3	3.3	20.0	0.0	80.0	0.0
Gm_AFGP4	37.5	34.4	21.9	0.0	3.1	3.1	0.0	30.0	0.0	23.3	20.0	16.7	0.0	10.0	16.1	0.0	80.6	3.2
Gm_AFGP5	44.9	31.9	18.1	0.0	2.9	0.0	2.2	25.4	1.4	35.5	24.6	5.1	0.0	8.0	21.7	0.0	78.3	0.0
Gm_AFGP6	29.9	48.9	1.5	0.7	0.0	5.8	13.1	49.3	0.0	0.7	0.0	49.3	0.7	0.0	28.8	5.5	64.4	1.4
**Gm Average**	**38.6**	**36.7**	**13.5**	**0.1**	**3.1**	**4.6**	**3.3**	**37.6**	**1.6**	**19.1**	**12.7**	**23.9**	**0.7**	**4.4**	**22.0**	**1.6**	**74.2**	**2.2**
Mt_AFGP1	36.9	31.3	29.5	0.0	0.0	1.8	0.6	16.4	1.8	0.0	71.0	7.3	0.0	3.6	23.6	0.0	63.7	12.7
Mt_AFGP2	32.3	41.3	24.6	0.6	0.0	0.6	0.6	32.7	0.0	0.0	57.1	10.2	0.0	0.0	13.1	1.3	79.1	6.6
Mt_AFGP3	26.8	40.0	1.0	0.0	32.2	0.0	0.0	61.8	8.7	0.0	0.0	27.6	1.9	0.0	47.8	7.7	42.0	2.4
**Mt Average**	**32.0**	**37.5**	**18.3**	**0.2**	**10.7**	**0.8**	**0.4**	**36.9**	**3.5**	**0.0**	**42.7**	**15.0**	**0.6**	**1.2**	**28.2**	**3.0**	**61.6**	**7.2**
